# The End-State Comfort Effect in 3- to 8-Year-Old Children in Two Object Manipulation Tasks

**DOI:** 10.3389/fpsyg.2012.00445

**Published:** 2012-10-29

**Authors:** Birgit Knudsen, Anne Henning, Kathrin Wunsch, Matthias Weigelt, Gisa Aschersleben

**Affiliations:** ^1^Developmental Psychology Unit, Saarland UniversitySaarbrücken, Germany; ^2^Department of Sport and Health, University of PaderbornPaderborn, Germany

**Keywords:** end-state comfort effect, anticipatory planning, child development, motor development, action effects to investigate end-state comfort performance

## Abstract

The aim of the study was to compare 3- to 8-year-old children’s propensity to anticipate a comfortable hand posture at the end of a grasping movement (*end-state comfort effect*) between two different object manipulation tasks, the bar-transport task, and the overturned-glass task. In the bar-transport task, participants were asked to insert a vertically positioned bar into a small opening of a box. In the overturned-glass task, participants were asked to put an overturned-glass right-side-up on a coaster. Half of the participants experienced action effects (lights) as a consequence of their movements (AE groups), while the other half of the participants did not (No-AE groups). While there was no difference between the AE and No-AE groups, end-state comfort performance differed across age as well as between tasks. Results revealed a significant increase in end-state comfort performance in the bar-transport task from 13% in the 3-year-olds to 94% in the 8-year-olds. Interestingly, the number of children grasping the bar according to end-state comfort doubled from 3 to 4 years and from 4 to 5 years of age. In the overturned-glass task an increase in end-state comfort performance from already 63% in the 3-year-olds to 100% in the 8-year-olds was significant as well. When comparing end-state comfort performance across tasks, results showed that 3- and 4-year-old children were better at manipulating the glass as compared to manipulating the bar, most probably, because children are more familiar with manipulating glasses. Together, these results suggest that preschool years are an important period for the development of motor planning in which the familiarity with the object involved in the task plays a significant role in children’s ability to plan their movements according to end-state comfort.

## Introduction

Adults typically grasp objects by anticipating what they are intending to do with that object. For example, when intending to get a drink, a glass that is placed upside-down on a table is first grasped thumb-down, rotated by 180°, and then placed thumb-up. That is, adults start the movement with an uncomfortable thumb-down grip, in order to end the movement in a comfortable thumb-up grip. This so-called end-state comfort effect has generally been taken as evidence for the influence of optimization constraints in motor planning in a variety of object manipulation tasks (e.g., Rosenbaum et al., 1990; Rosenbaum and Jorgensen, 1992; Fischman, 1997, 1998; for a recent overview see Rosenbaum et al., 2012). In contrast to adults’ success in manipulating different objects according to end-state comfort, findings were inconsistent with regard to children’s performance as a function of age and type of task. Therefore, the general aim of the present study was to investigate end-state comfort performance in children across different ages in two different object manipulation tasks.

Most previous studies applied only a single task, specifically the bar-transport task, in order to investigate the end-state comfort effect in children. For example, in a study conducted by Manoel and Moreira (2005) 3- to 6-year-old children had to insert either the right or the left end of a horizontally resting bar into a box with either a cylindrical hole (low-precision condition) or with a semi-cylindrical hole (high precision condition). In both conditions, a right-end insertion required a comfortable overhand grip (uncritical trials), whereas a left end insertion required an uncomfortable underhand grip (critical trials) for right-handed children. Results revealed little evidence of end-state comfort, independent of age, and condition, with even the oldest children tending to grasp the bar with an overhand grip in the majority of cases, even if this meant to finish the maneuver in an uncomfortable posture. However, using a similar bar-transport task with 3- to 5-year-old children, Weigelt and Schack (2010) found an increase in end-state comfort performance from 18% in the 3-year-olds to 70% in the older children (see also Stöckel et al., 2011, for evidence of an increase of end-state comfort performance from 50 to 92% in 7- to 9-year-old children). Thibaut and Touissant (2010) also used the bar-transport task in 4-, 6-, 8-, and 10-year-old children. Whereas end-state comfort performance rose from 40% in the 4-year-olds to 70% in the 6-year-olds, performance dropped in the 8-year-olds to 50% and rose again to 80% in the 10-year-olds. Interestingly, when the bar was replaced by a two-colored pencil, and children were asked to pick up the pencil to trace an alley on a sheet of paper (high precision requirements), the 8-year-olds now performed better than the 6-year-olds. This suggests that precision requirements together with the familiarity of the object involved in the task might have helped these children to plan their movements more efficiently. Adalbjornsson et al. (2008) also investigated end-state comfort performance in a task that involved familiarity with the object used. They employed the overturned-glass task by asking two groups of preschool children (2–3 years and 5–6 years) to pick up an upside-down glass and to pour water into it, both with their preferred hand. However, only 20% of the 2- to 3-year-olds and 35% of the 5- to 6-year-olds grasped the glass according to end-state comfort.

Although, these studies generally suggest an increase in end-state comfort performance with age, they also show that the propensity to use end-state comfort in object manipulation tasks differs in children of comparable age within and across tasks. For example, whereas Manoel and Moreira (2005) found only little evidence of the end-state comfort effect in 6-year-old children in the bar-transport task, children of the same age showed end-state comfort 70% of the time in a bar-transport task as reported by Thibaut and Touissant (2010). Likewise, whereas Weigelt and Schack (2010) found 70% end-state comfort performance in 5-year-old children in the bar-transport task, Adalbjornsson et al. (2008) found only 35% end-state comfort performance in 5- to 6-year-old children in the overturned-glass task. These conflicting results might be due to differences within the tasks, such as precision requirements and task demands, as well as differences across tasks, such as familiarity with the object to be manipulated. Therefore, it would be interesting to compare children of the same age for two different tasks within one single study. To the best of our knowledge, this was only done once before by Smyth and Mason (1997), who examined the performance of children from 4 to 8 years of age in the bar-transport task and the handle-rotation task. The latter required children to rotate a handle on a disk in order to cover pictures printed at different degrees on the disk (see also Crajé et al., 2010; van Swieten et al., 2010; for different versions of the handle-rotation task with children). According to the results, however, the end-state comfort effect was not present even in the oldest children, no matter which task was used. Hence, it is still an open question whether the presence of the end-state comfort effect differs between two tasks for children of the same age. If one would find different developmental patterns of the end-state comfort effect between tasks, then this would be evidence for the strong role of task constraints on the emergence of anticipatory planning skills in young children.

Another factor that might lead to different results in end-state comfort performance in young children might be the particular set-up used. Young children might find it easier to plan their actions according to end-state comfort, if their movements lead to interesting effects in the environment, such as a light that turns on as a consequence of their movement. This was the case in a recent study by Jovanovic and Schwarzer (2011), who used a modified version of the bar-transport task with 18-, 24-, and 42-month-old children. Instead of presenting the bar horizontally, the bar used by Jovanovic and Schwarzer had a small platform on one end, which held the bar in a vertical position. By way of demonstration, the bar stood with its platform on its top (requiring a comfortable thumb-up grip) and children were shown that when the bar was inserted into the cylinder, lights lit up that were built into the cylinder. The experimenter modeled the thumb-up grip twice for the child (*baseline condition*) and subsequently, the bar was returned to its starting position and the child was encouraged to perform the same action as the experimenter. Then, the bar was returned to its starting position, but this time standing on its platform (*reverse condition*) and children were asked to switch on the lights (now requiring an uncomfortable thumb-down grip). Only 8% of the 18-month-olds and none of the 24-month-olds grasped the bar with an uncomfortable thumb-down grip. In contrast, a comparatively high percentage of 60% of the 42-month-olds showed the end-state comfort effect. From the latter observation, the question arises whether the high percentage of children showing the end-state comfort effect at this age is a result of the action effects presented at the end of the object manipulation.

There is ample evidence, that action effects, such as lights (Paulus et al., 2011), sounds (Hauf et al., 2004; Paulus et al., 2012), or both (Elsner and Aschersleben, 2003; Hauf and Aschersleben, 2008) play an important role in how infants control their actions. According to the common coding theory, which is based on the ideomotor theory proposed by James (1890), actions are planned and selected by anticipating the corresponding action effects (Prinz, 1997; Hommel et al., 2001). Through repeated co-occurrences of particular actions and their effects, action–effect associations are established. Planning an action is therefore assumed to activate the representation of the desired action effect (e.g., making a light occur), which then results in a priming of the corresponding movement (e.g., pressing a button; Kunde, 2001; Pfister et al., 2010). Action-effect associations can either be learned by ways of instrumental learning (e.g., DeCasper and Fifer, 1980; Elsner and Hommel, 2004) or by observation (e.g., Elsner and Aschersleben, 2003; Paulus et al., 2011). In the context of observational learning of action-effect associations it is not only necessary to represent the particular action-effect, but also to relate the other’s action to one’s own motor repertoire. Elsner and Aschersleben (2003) have shown that 15-month-old infants indeed already expect their own actions to produce the same effects as the observed action. Similarly, if 14-month-old infants see a model touch a lamp with her forehead, they imitate this action significantly more often if it was followed by a light effect than when it was not (Paulus et al., 2011).

However, although in the study reported by Jovanovic and Schwarzer (2011) action effects were involved, the experimental situation was somewhat different to the typical set-up used in the imitation studies testing the role of action effects reported above. First of all, the lights always lit up when the bar was inserted into the cylinder. That is, the same action effect always followed the action, irrespective of the grip selected. Moreover, in the *reverse condition* tested by Jovanovic and Schwarzer (2011), a thumb-down grip was never demonstrated to the children. Instead, children where only shown the starting state (bar resting on its platform) and the end-state (bar in the lit cylinder; shown in the preceding baseline condition) and children had to infer the movement in order to switch on the lights. That is, rather than being able to rely on established action-effect associations, children in this study had to emulate the action necessary to reach the goal. In contrast to goal imitation, goal emulation has been described as being a case where an observer attempts to reproduce a completed goal (e.g., bar in a lit cylinder) by whatever means seem suitable, without having observed the exact action used by the actor to reach the goal (Tomasello, 1999). Studies on goal emulation in infancy suggest, that the ability to make inferences from the observed goal to the required movement emerges by the end of the second year (e.g., Bauer et al., 1999; Huang et al., 2002; see Elsner, 2007, for a review of the role of movements and their effects in infants’ emulation of goal-directed actions). Even though the common coding theory does not make predictions about the influence of action effects on goal emulation, it is still possible that the light effects used in the study by Jovanovic and Schwarzer (2011) did help the 42-month-old children to plan their movements more efficiently by indirectly enhancing children’s motor planning by, for example, affecting attentional or motivational processes. That is, the light effects might have rendered the goal more salient and therefore the light effects might have motivated children to accomplish the task more accurately.

### The present study

The first aim of the present study was to compare the presence of the end-state comfort effect in children of different ages between two different object manipulation tasks. The second aim was to investigate, whether the light effects in the study by Jovanovic and Schwarzer (2011) was the determining factor with regard to the comparatively high percentage of end-state comfort shown by the 42-month-old children. To this end, we investigated end-state comfort performance in six age groups of children from 3 to 8 years, as well as a control group of adults, in the bar-transport task following Jovanovic and Schwarzer (2011) and in a version of the overturned-glass task adapted from Adalbjornsson et al. (2008). In both tasks, half of the participants in each age group experienced action effects as a consequence of their movements (AE groups) while the other half of the participants did not (No-AE groups). In neither of the two tasks was a thumb-up or a thumb-down grip demonstrated to the participants. Based on the literature discussed above, we expected to find a general increase in end-state comfort performance across age in both tasks. In addition, if the presence of an interesting action effect was the determining factor with regard to the comparably high percentage of end-state comfort shown in children aged 3–4 years in the study by Jovanovic and Schwarzer (2011), especially children at the younger ages, during which end-state comfort is still developing, should benefit from the presence of an action effect.

## Materials and Methods

### Participants

Six age groups of 16 participants each and an adult control group (*n* = 20) took part in the study (3-year-olds: nine female, *M* age = 41.6 months, SD = 2.87, 15 right-handed; 4-year-olds: five female, *M* age = 55.6, SD = 2.55; 15 right-handed; 5-year-olds: eight female, *M* age = 66.2, SD = 3.31, all right-handed; 6-year-olds: eight female, *M* age = 75.5, SD = 2.89, 13 right-handed; 7-year-olds: nine female, *M* age = 89.2, SD = 3.21, all right-handed; 8-year-olds: seven female, *M* age = 102.44, SD = 3.21, 13 right-handed; and adults: 11 female, *M* age = 25.6 years, SD = 5.2, 18 right-handed). Participants were recruited and tested in different kindergartens in the Saarbrücken area of Germany and in the Developmental Psychology Unit, Saarland University, Germany. The adult control group was not included in the analyses, because all of the participants showed the end-state comfort effect in both tasks (see Thibaut and Touissant, 2010, for similar results). Ten additional children were tested, but their data were excluded from further analyses, because they did not understand German (one 3-year-old) or did not understand the task (one 3-year-old), due to an experimenter error (three 3-year-olds, two 4-year-olds, one 5-year-old), or because the child was unwilling to finish the task (two 3-year-olds). In each age group, there were eight children in the AE group and eight children in the No-AE group, except for the 7-year-old group with nine children in the AE group and seven children in the No-AE group.

### Apparatus

**Figure 1:**
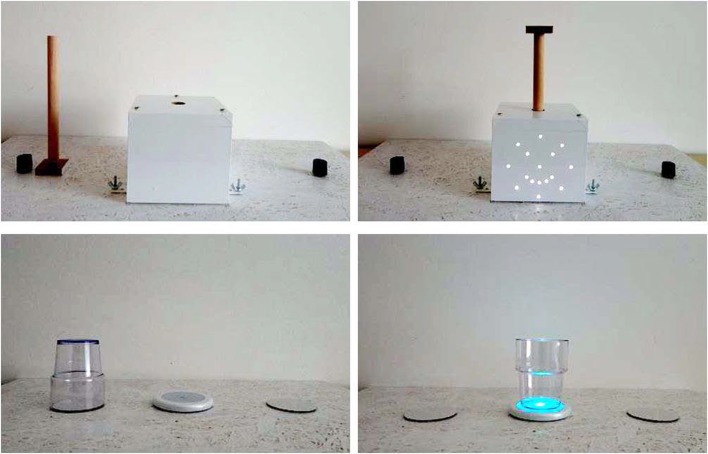
**Left column: starting position of the bar and the glass in the critical trial in the bar-transport task and right-hand-trial in the overturned-glass task**. Right column: final position of the bar and the glass for the AE groups in the bar-transport task and the overturned-glass task.

In both tasks, the bar-transport task and the overturned-glass task, materials were placed on a white wooden board (40 cm × 66 cm) on a table. A starting line was marked on the floor at approximately 70 cm in front of the table. The material of the bar-transport task consisted of a white box (13 cm × 13.5 cm × 11.5 cm) with an insertion hole (diameter: 3- to 6-year-olds: 2 cm, 7- and 8-year-olds: 2.5 cm, adults: 3 cm) on its top and a smiley configuration of LEDs inserted in its front side facing the child. Twenty-three centimeters to the left and to the right of the box a bar holder was placed, which held the bar in an upright position. Pilot data had revealed that mainly the young children were uncomfortable with handling a rather thick bar. Therefore, the bar used (and the corresponding hole in the box) was of different size for the kindergarten children, the school children, and the adults, in order to adjust for different hand sizes and to ensure precision requirements (3- to 6-year-olds: diameter bar = 1.6 cm, platform: 4 cm × 4 cm; 7- and 8-year-olds: diameter bar = 2 cm, platform: 4.5 cm × 4.5 cm; adults: diameter bar = 2.5 cm, platform: 5 cm × 5 cm; bar length: all 20 cm). For the AE groups, a point-light-smiley lit up on the front of the box when the bar was inserted (see Figure 1, top right). The point-light-smiley consisted of 16 LED lights (Homefit lightning, 20 LEDs, 3.3V/0.066W) arranged in an outer circle (diameter: 8.6 cm) of 8 LEDs with a distance of 3.2 cm between each LED light and additionally, 2 LEDs for the eyes (distance: 3.4 cm), 1 for the nose and 5 for the mouth (distance: 1 cm).

The material used in the overturned-glass task consisted of an OSRAM LUX pod coaster (outer diameter: 9 cm; inner diameter: 6.5 cm) and a transparent, plastic glass (height: 9.5 cm, diameter: 5.5 cm on the bottom, and 7.5 cm on the top). The glass could be grasped near its narrow bottom by children with small hands. Alternatively, the glass could be grasped near its wide end by children with comparatively bigger hands, therefore adjusting for different hand sizes of the different age groups. Note, that in this task precision requirements were comparable to the bar-transport task, since the diameter of the bottom of the glass just fitted the inner diameter of the coaster. A white cardboard circle (diameter: 6 cm) was glued on the board 23 cm centimeters to the left and to the right of the coaster, in order to keep the starting position constant and to prevent the glass from slipping when being grasped. For the AE groups, the coaster lit up when the glass was placed on top of it (see Figure 1, bottom right). In contrast, for the No-AE groups, the batteries were removed in both set-ups and light effects never occurred. A camera was positioned behind the table, facing the participant, and all sessions were videotaped for future reference.

### Tasks and procedure

Participants were tested individually with three experimenters in the room. Only Experimenter 1 interacted with the participant and gave instructions. Experimenter 2 prepared the set-up between trials and tasks and Experimenter 3 took note of the participant’s grip. Since adults follow instructions more readily, they were tested with only two experimenters in the room. Both tasks consisted of six trials and each trial began with the participant standing at the starting line. Before the first trial of each task, the starting state (bar resting on its platform, upside-down glass; see Figure 1, left column) and the desired end-state (bar in the box, glass on the coaster, see Figure 1, right column) was shown to the participant by Experimenter 2. However, how Experimenter 2 grasped the bar/glass was never demonstrated to the participant, neither during the demonstration nor before the first trial or in between trials. Experimenter 2 always covered the set-up with her body, and additionally, she covered her movements with a clipboard when grasping and moving the bar/glass. The starting position of the bar/glass was always opposite to the participants’ to-be-used hand (e.g., for a right-hand-trial the bar/glass was placed to the left of the box/coaster). This was done in order to keep the movement required to grasp the bar/glass (moving the arm diagonally across the body’s midline) constant across both tasks.

### The bar-transport task

In the bar-transport task, participants were asked to insert the bar into the opening of the box with their preferred hand and to put the non-preferred-hand behind their back. The bar-transport task was always performed with the preferred hand. Half of the trials were critical trials, which started with the bar being placed on its platform next to the bar holder (see Figure 1, top left). In critical trials, a thumb-down grip was required, followed by a 180° rotation to end in a comfortable thumb-up position. The other half of the trials were uncritical trials, which started with the bar being placed in the bar holder, requiring a thumb-up grip with no rotation of the bar.

### The overturned-glass task

In the overturned-glass task participants were asked to put the glass right-side-up on the coaster. In order to see if handedness has an impact on end-state comfort performance, half of the trials were preferred-hand-trials, in which participants had to use their preferred hand and to put their non-preferred-hand behind their back. The other half of the trials were non-preferred-hand-trials, in which participants had to use their non-preferred-hand and to put their preferred hand behind their back. In both, preferred- and non-preferred-hand-trials, a thumb-down grip of the glass was required followed by a 180° rotation of the glass to reach end-state comfort. In both tasks, if the child had difficulties using only one hand, the experimenter took the child by their not-to-be-used hand, walked them to the table, and kept hold of their hand until they had completed the trial.

For each age group, the order of the tasks was counterbalanced. That is, half of the participants received the bar-transport task first and the other half of the participants received the overturned-glass task first. The trial order of the two sorts of trials in each task (critical/uncritical in the bar-transport task; preferred/non-preferred-hand in the overturned-glass task) was randomized such that (a) half of the participants started the task with a critical (preferred hand) trial and the other half of the participants started the task with an uncritical (non-preferred-hand) trial (b) the same sort of trial was administered maximally two times in a row. A trial was repeated if (a) both hands were used, (b) the wrong hand was used, (c) the glass/bar was grasped on its top, (d) if the glass was not turned, (e) if the glass/bar was turned on the participant’s chest, or (f) an experimenter error occurred (such as indicating the wrong hand). Handedness of all child participants was determined before the start of the experiment by registering the participant’s preferred hand when throwing a ball, holding a spoon, and drawing a face (one trial per task). The participant’s preferred hand was determined by the hand that was used in at least two out of the three activities.

### Coding

For each trial, participants’ grip was coded. In the bar-transport task, the score 1 was given if participants grasped the bar thumb-down followed by a 180° rotation in critical trials and if participants grasped the bar thumb-up followed by no rotation in uncritical trials. The score 0 was given in all other cases. In the overturned-glass task, score 1 was given if participants grasped the glass thumb-down followed by a 180° rotation. The score 0 was given in all other cases. In accordance with Adalbjornsson et al. (2008) and Weigelt and Schack (2010), the end-state comfort effect was considered to be present if the score 1 was given in at least two out of three trials. All six trials of a randomly chosen set of 25% of the participants of each age group were coded by a second coder, blind to hypotheses of the study. Inter-rater reliability was perfect, Cohen’s κ = 1.

## Results

In the following, the results regarding children’s performance in the presence or absence of an action effect are reported first. Then, the results on the influence of age on end-state comfort performance are reported for (1) the bar-transport task, (2) the overturned-glass task, and (3) the comparison between the two tasks. For all analyses, non-parametric tests were used with a significance level of α = 0.05 and with *p*-values between 0.05 and 0.10 considered as marginally significant. All *p*-values reported are two-tailed.

### Action effects

There was no difference in end-state comfort performance between the AE and the No-AE groups in critical trials in the bar-transport task (Chi-square exact, *p *= 0.394; Fisher’s exact tests per age group, all *p*s > 0.467) and neither in preferred-hand-trials (Chi-square exact, *p* = 0.326, Fisher’s exact tests per age group, all *p*s > 0.585) or in non-preferred-hand-trials (Chi-square exact, *p* = 1, Fisher’s exact tests per age group, all *p*s > 0.438) in the overturned-glass task (see Table 1). For the following analyses we therefore pooled the data of the AE groups and the No-AE groups in both tasks.

**Table 1:** **Percentages of end-state comfort in action effect (AE) groups and no-action effect (No-AE) groups in the bar-transport task and the overturned-glass task**.

Age (years)	Bar-transport task				Overturned-glass task			
	Critical		Uncritical		Preferred		Non-preferred	
	AE	No-AE	AE	No-AE	AE	No-AE	AE	No-AE
3	0	25	100	75	75	50	75	63
4	38	38	88	100	75	63	63	75
5	75	88	100	100	75	88	100	100
6	75	75	88	100	88	75	88	75
7	86	89	86	86	86	86	86	100
8	88	100	100	88	100	100	100	88

*All percentages are based on *n* = 8, with an except for the 7-year-olds (AE group *n* = 9, No-AE group *n* = 7)*.

### Influence of age on end-state comfort performance

#### Bar-transport task

**Figure 2:**
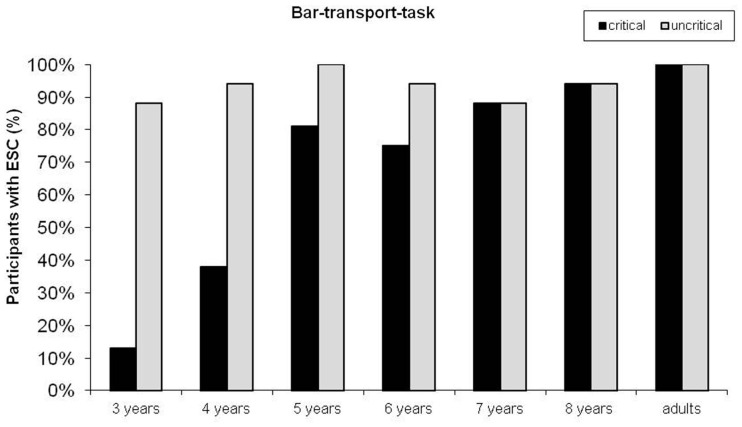
**Percentages of participants showing the end-state comfort effect (ESC) in critical and uncritical trials across age in the bar-transport task**.

The percentage of participants using a thumb-down grip in critical trials and the percentage of participants using a thumb-up grip in uncritical trials (in at least two out of three trials) in the bar-transport task for each age group are depicted in Figure 2. Almost all participants in all age groups grasped the bar thumb-up in uncritical trials with no significant difference between the age groups (Chi-square exact, *p* = 0.93). However, in the critical trials end-state comfort performance differed significantly between the age groups (Chi-square exact, *p* < 0.001) and increased with age: 3-year-olds 13%, 4-year-olds 38%, 5-year-olds 81%, 6-year-olds 75%, 7-year-olds 88%, 8-year-olds 94%. When compared separately, end-state comfort performance was significantly different between the 3- and the 5-, 6-, 7-, and 8-year-olds (Fisher’s exact, all *p*s < 0.001), the 4- and the 5-, 7-, and 8-year-olds (Fisher’s exact test, *ps *< 0.029). There was no effect of task order (bar-transport task first or second; Fisher’s exact test over all groups, *p* = 0.135, Fisher exact tests per age group, all *p*s > 0.262), trial order (critical trials first or second; Fisher’s exact test, *p *= 0.832), or gender (Fisher’s exact test, *p* = 0.202) on end-state comfort performance in critical trials.

Trial repetitions were unlikely to influence end-state comfort performance. In each age group, participants performed a total of 96 trials and the total number of trial repetitions per age group was 25 for 3-year-olds, 19 for 4-year-olds, and 7 for each of the remaining older age groups.

#### Overturned-glass task

**Figure 3:**
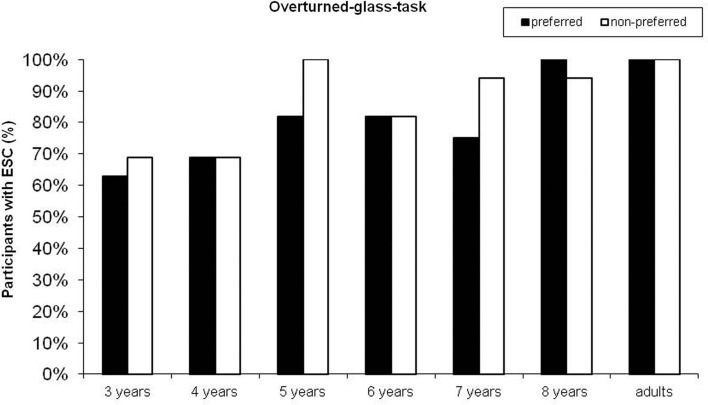
**Percentages of participants showing the end-state comfort effect (ESC) in preferred hand and non-preferred-hand-trials across age in the overturned-glass task**.

As depicted in Figure 3, the percentages of end-state comfort performance in preferred-hand-trials and in non-preferred-hand-trials did not differ significantly in neither of the age groups (McNemar, all *p*s > 0.250). In the following only analyses on preferred-hand-trials are reported in order to directly compare children’s performance in the two tasks.

There was a significant increase in end-state comfort performance with age (Chi-square exact, *p* = 0.006): 3-year-olds 63%, 4-year-olds 69%, 5-year-olds 82%, 6-year-olds 82%, 7-year-olds 75%, 8-year-olds 100%. When compared separately, end-state comfort performance was significantly different between the 3- and the 8-year-olds (Fisher’s exact test, *p* = 0.018) and the 4- and the 8-year-olds (Fisher’s exact test, *p* = 0.043). There was no effect of task order (overturned-glass task first or second; Fisher’s exact test over all groups, *p* = 0.622, Fisher’s exact tests per age group, all *p*s > 0.550), trial order (preferred-hand-trials first or second; Fisher’s exact test, *p* = 1), or gender (Fisher’s exact test, *p* = 0.34) on end-state comfort performance.

Trial repetitions were unlikely to influence end-state comfort performance. Per age group participants performed a total of 96 trials and the total number of trial repetitions per age group was 26 for 3-year-olds, 12 for 4-year-olds, 11 for 5-year-olds, 12 for 6-year-olds, 11 for 7-year-olds, and 11 for 8-year-olds.

In order to investigate whether children may have learned to grasp the glass according to end-state comfort across trials, for each child, trial scores were summed across the first half (trials 1–3) and across the second half (trials 4–6) of the task, regardless of trials being performed with the preferred- or non-preferred-hand. When comparing children’s end-state comfort performance in the first half with the second half of the task, there was no indication of learning (Wilcoxon, *p* = 0.16).

#### Performance across tasks

When comparing end-state comfort performance between critical trials in the bar-transport task and preferred-hand-trials in the overturned-glass task, differences were statistically significant only for the 3-year-olds (McNemar, *p* = 0.008), marginally significant for the 4-year-olds (McNemar, *p* = 0.063), and not significant for the other age groups (all *p*s > 0.625). Accordingly, a higher number of 3-and 4-year-olds showed the end-state comfort effect in the overturned-glass task, as compared to the bar-transport task.

## Discussion

The first aim of the present study was to compare the presence of the end-state comfort effect in children of different ages between two different object manipulation tasks, the bar-transport task, and the overturned-glass task respectively. In line with Weigelt and Schack (2010) and Stöckel et al. (2011), we found an increase in end-state comfort performance in the bar-transport task. Accordingly, children’s propensity to use an uncomfortable thumb-down grip in critical trials rose from 13% in the 3-year-olds to 94% in the 8-year-olds. Interestingly, the number of children showing the end-state comfort effect in this task doubled from 3 to 4 years and from 4 to 5 years of age, whereas the older age groups differed only slightly in end-state comfort performance. This might suggest that the age between 3- and 5 years is an important period, in which children progressively become better in planning their movements.

However, when examining children’s performance in the overturned-glass task, a different pattern of results was found. Here, end-state comfort performance also increased with age, but in contrast to the findings of Adalbjornsson et al. (2008), already 63% of the 3-year-olds in the current study grasped the glass according to end-state comfort. Thus, whereas only 13% of the 3-year-olds showed the end-state comfort effect in the bar-transport task, 63% of the 3-year-olds showed the end-state comfort effect in the overturned-glass task. This difference in end-state comfort performance at the younger ages might be explained by the child’s familiarity with the object involved. The child’s familiarity with the object involved pertains to the amount of prior experience children have gathered with that object throughout lifetime. Object manipulations with every day objects, such as glasses, are likely more familiar to preschoolers than object manipulations with novel objects, such as the bar used in the present study. For example, studies investigating tool-use in young children show that, around 1 year of age, the way children grasp objects is influenced by the intended future use. This is evidenced by distinct movement kinematics for actions on different objects (Claxton et al., 2003) or progressively more efficient grasping strategies used for a familiar tool, such as a spoon containing food across 9-, 14-, and 19-month-old children (McCarty et al., 1999). More specifically, Barrett et al. (2007) have shown that the familiarity with a tool (prior experience) influences its use in a novel task in 12- and 18-month-old children. In their study, children had to turn on a light inside a box by using either a familiar tool (spoon) or an unfamiliar tool (spoon-like object). In one condition, both tools fit in the box with their handle end only. Results revealed that children tended to grasp the familiar spoon by its handle even though children were shown to grasp the spoon by its bowl end for insertion. In contrast, the unfamiliar spoon was grasped much more flexibly and led to significantly more successes. Thus, children’s familiarity with the object manipulations involved in this novel task likely biased their tool-directed actions.

This bias might be explained by a competition between the goal-directed and the habitual system as suggested by Herbort and Butz, 2011; see also Stöckel et al., 2011 for a similar interpretation). The goal-directed system selects grasping movements according to the intended future use of the object (insertion into the box), whereas the habitual system selects grasping movements that are habitually used to grasp the object (self-feeding). When presented with an unfamiliar tool, both systems select the same action (insertion into the box). However, when presented with a familiar tool, the habitual system trumps the goal-directed system and a grasping movement that has been used repeatedly in the past for that object is selected (self-feeding). Therefore, in the study by McCarty et al. (2001), the habitual system likely had hindered children in solving a novel task with a familiar object. In contrast, in the present study, the habitual system might have helped children in solving a familiar task (overturned-glass task) with a familiar object. That is, the greater familiarity with handling glasses in the present study might have helped children to plan their movements in the overturned-glass task more accurately as compared to the bar-transport task. In the bar-transport task, the habitual system likely did not help children in solving the task, since children may not have gathered sufficient experience with the object manipulations required. Indeed, when comparing end-state comfort performance between critical trials in the bar-transport task and preferred-hand-trials in the overturned-glass task, 3- and 4-year-old children were better in manipulating the glass according to end-state comfort in the overturned-glass task than they were able to manipulate the bar in the bar-transport task. The older children, in contrast, were able to manipulate both, the glass and the bar equally efficient. Therefore, when considering children’s performance in both tasks, results show that the age between 3- and 5 years is an important period for motor planning in which the familiarity with the object involved in the task may play an important role in children’s ability to plan their movements according to end-state comfort.

Given this finding, the question arises whether the bar-transport task and the overturned-glass task were truly comparable, since in the bar-transport task an uncomfortable thumb-down grip was required only in half of the trials, whereas in the overturned-glass task an uncomfortable thumb-down grip was required in all the six trials. Consequently, when considering that children in the overturned-glass task were given the opportunity to use end-state comfort twice as much as compared to the bar-transport task, children might have learned across trials to use a thumb-down grip. If so, one might expect children to perform better in the bar-transport task when the latter was administered after the overturned-glass task. However, there was no order effect of tasks found. Furthermore, when comparing end-state comfort performance across the first and second half of trials in the overturned-glass task, there was no indication of learning, suggesting that indeed familiarity with the object has a significant impact on young children’s propensity to use end-state comfort in object manipulation tasks. This interpretation is supported by a recent study showing that preschoolers imitate familiar tool-use actions more correctly than unfamiliar tool-use actions (Wang et al., 2012).

It could also be argued that anticipatory motor planning for manual action is object or task specific for known objects or tasks, especially for younger children. This can be inferred from the different onsets of the end-state comfort effect for the two different tasks, the bar-transport task and the overturned-glass task, respectively. Younger children were more proficient in solving the overturned-glass task than the bar-transport task. This shows that the end-state comfort effect does not generalize simply from one task to another task. Importantly, adding an action effect at the end of the manipulatory maneuver did not make the end-state comfort effect to occur more often. This is an interesting aspect, since it has been shown elsewhere that action effect associations are formed by children of the same age or even younger (Verschoor et al., 2010). For example, Eenshuistra et al. (2004) showed that already 4-year-old children are able to acquire response-effect associations. At the same time, these children still display stronger stimulus-driven behavioral tendencies as compared to 7-year-old children, and they are less able to maintain the task goal. Thus, it seems reasonable to assume that anticipatory planning skills develop at different rates regarding object manipulation and the acquisition of response-effect associations. This assumption should be tested in future studies.

The second aim of this study was to investigate whether the action effects presented in the bar-transport task by Jovanovic and Schwarzer (2011) were the determining factor with regard to the comparatively high percentage of 60% end-state comfort shown by the 42-month-old children. The present results suggest that it is unlikely that the high percentage of end-state comfort performance of the 42-months-old children reported by Jovanovic and Schwarzer (2011) can be attributed to the presence of action effects. Children in the present study did not benefit from the action effects presented, neither in the bar-transport task nor in the overturned-glass task. This is most likely explained by one important aspect that makes this study different from other studies investigating the influence of action effects in infants (e.g., Hauf et al., 2004; Paulus et al., 2011). Children were never shown the uncomfortable thumb-down grip in order to make the light effects occur and, consequently, no action effect associations could have been formed by observation. Instead, children were only shown the starting state and the end-state of the action, without the corresponding action and children had to emulate, rather than imitate, the action. According to the common coding theory, however, the formation of action effect associations is crucial. Only through repeated co-occurrences of the uncomfortable thumb-down grip and the following light effect could children have associated the light effect with a thumb-down grip and hence, anticipating the light effect might have helped them plan their movements more readily according to end-state comfort. In addition, the light effects were presented independent of the initial grasp used by the child (thumb-down or thumb-up). That is, also thumb-up grips in critical trials (and in preferred- or non-preferred-hand-trials) were followed by a light effect. Consequently, one might expect that children in the AE groups, who used a comfortable thumb-up grip in the first critical trial will use a comfortable thumb-up grip in the following trials, independent of trial type, due to instrumental learning established by the rewarding light effect (smiley). However, inspection of the data revealed that all children changed grip posture in either task with an increase in thumb-down grips across age. Even in the 3-year-olds each child showed an uncomfortable thumb-down grip in the overturned-glass task at least once.

Still, it may be that the presence of an action affect may indirectly enhance young children’s performance in these tasks by positively affecting their interest in performing the task. Already very young infants easily detect a contingent relation between their own movements and subsequent effects and greatly enjoy this experience of self-efficacy (Watson, 1972). Thus, the relatively simple and possibly boring actions of inserting a bar into a hole and putting a glass on a coaster may become more interesting once they are instrumental to self-produce a more interesting event. In a similar vein, the presence of an action effect adds a reason to performing the action itself, distinct from simple compliance with the experimenter’s instructions. As there seems to be a general teleological bias in human reasoning, that is, a tendency to ask what objects and events *are for* (Kelemen, 1999), qualifying an action as a mean to achieve a goal might indirectly enhance children’s motor planning in end-state comfort tasks by affecting, for example, attentional or motivational processes. In this sense, it might still have been possible that the comparatively high percentage of end-state comfort performance of the 42-month-old children reported by Jovanovic and Schwarzer (2011) was due to such attentional or motivational factors. The current findings, however, suggest that the action effects used by Jovanovic and Schwarzer did not play a role in end-state comfort planning, neither directly according to the principles of the common coding theory, nor indirectly via enhancing children’s interest in performing the task.

Even though the present findings are in line with previous studies that found a general increase in end-state comfort performance over age, the results of this study are opposite to findings reported by Adalbjornsson et al. (2008) and Jovanovic and Schwarzer (2011). Adalbjornsson et al. (2008) found only little evidence for end-state comfort in the overturned-glass task in 2- to 6-year-old children, whereas in the present sample already 63% of the 3-year-olds grasped the glass according to end-state comfort. This discrepancy might be due to differences in the experimental set-up. In the study by Adalbjornsson et al. (2008) children were sitting in front of the table during test trials. This might have caused some motorical restrictions whereas in the actual study, children were standing during testing, and could therefore move their arms more freely. In addition, Adalbjornsson et al. (2008) asked the children not only to turn the glass but also to pour water from a pitcher into it. For both action parts, children were only allowed to use their preferred hand. Thus, children were not only asked to perform a rather complex action sequence, it might also be that the planning of the second action (grasping the pitcher by using a thumb-up grip and pouring water into the glass) influenced the way the first action (turning the glass) is performed. This explanation is supported by studies showing that later elements of an action sequence are already planned and specified at the beginning of the sequence (see, e.g., Inhoff et al., 1984).

Likewise, in the bar-transport task conducted by Jovanovic and Schwarzer (2011) already 60% of the 42-month-olds grasped the bar according to end-state comfort, whereas only about half as much 3- and 4-year-old children in the present study showed the end-state comfort effect in the bar-transport task. Although tasks were quite similar, still some differences in the procedure of the two studies might account for this inconsistent result. In the bar-transport task with 42-month-old children (Experiment 1) of Jovanovic and Schwarzer (2011) no care was taken to occlude the rotation of the bar when the experimenter returned the bar to its starting position in between trials. Even though 18- and 24-month-old children did not benefit from observing the experimenter performing the transport in the reverse condition as shown in Experiment 3 of the same study, the 42-month-olds tested in Experiment 1 might have benefited from observing the experimenter grasping the bar according to end-state comfort when returning the bar. In contrast, children in the current study never saw the experimenter grasping the bar.

There is also a notable difference in end-state comfort performance in the bar-transport task of children of comparable age found by Stöckel et al. (2011) and the present study. Stöckel et al. (2011) found that 50% of the 7-year-olds, 67% of the 8-year-olds, and 92% of the 9-year-olds used end-state comfort. In contrast, in the present study, comparable success rates shifted toward the younger age groups with already 81% of the 5-year-olds showing the end-state comfort effect. One possible explanation for this shift of success rate in age may be the different rotations of the bar required in each task. Whereas the bar-transport task by Stöckel et al. (2011) required only a 90° rotation, the bar-transport task in the present study required a 180° rotation. If the bar has to be rotated by 180° an initial comfortable grip would end in an even more uncomfortable grip (arm rotated counterclockwise 180° rather than 90°) for a 180° rotation than for a 90° rotation, which children might have sought to avoid. However, in the bar-transport task reported by Weigelt and Schack (2010) also only a 90° rotation of the bar was required and their results are comparable to the results of the present study (18% end-state comfort in 3-year-olds, 47% in 4-year-olds, and 70% in the 5-year-olds). This suggests that other differences between the studies are more likely to account for the difference in end-state comfort performance shown by children of comparable age.

There are several methodological differences between the present study and previous work that might account for the inconsistent findings reported on the development of the end-state comfort effect in young children. These differences relate, for example, to the particular task and the version of the task used, the familiarity of the object to be manipulated, task complexity, and precision requirements. In addition, also the specific procedure used in a study might influence children’s performance in motor planning tasks. For example, it might make a difference, if participants are standing or sitting when performing the task. In the majority of studies that found the end-state comfort effect in children, including the present work, the children were standing during testing and could therefore move their arms more freely (e.g., Weigelt and Schack, 2010; Stöckel et al., 2011). In contrast, in several studies that did not observe the end-state comfort effect, children were sitting during the testing session (e.g., Manoel and Moreira, 2005; Adalbjornsson et al., 2008; van Swieten et al., 2010).

Finally, the development of different cognitive abilities, such as executive functions, might explain some of the inconsistencies in the findings. It is interesting to note that around the same time during the preschool years when end-state comfort planning seems to develop, children also show a marked improvement in higher level cognitive processes, termed executive function, that are involved in planning and controlling goal-directed behavior (Zelazo et al., 1997). Therefore, differences in task demands that are due to differences in methodology, such as those mentioned above, may have an effect on task performance. This is especially true for the preschool age, which seems to be a period of marked development in end-state comfort planning. For example, the less complex a given task, the less executive control it may require, or the more familiar the child is with the to be manipulated object, the more cognitive resources may be available for motor planning and control. It is therefore possible, that differences in task demands which tap into executive control processes may have a greater effect on younger compared to older children’s performance in end-state comfort tasks. Further research is needed to investigate whether executive function skills play a role in the development of end-state comfort planning.

To summarize, the current work provides three major findings. First, despite inconsistencies in the literature regarding the onset of end-state comfort planning in childhood and its prevalence at specific ages, several studies point to a general increase in motor planning skills as indicated by the end-state comfort effect across the preschool and school years. The results of the present study are in line with this observation. Second, results suggest that the high percentage of end-state comfort performance of the 42-months-old children reported by Jovanovic and Schwarzer (2011) cannot be attributed to the presence of action effects. Third, the comparison of two different object manipulation tasks within the same participants allowed us to investigate the influence of the particular task used as a possible contributing factor to the inconsistent results found across studies. Results suggest that the familiarity with the object involved in the task does play a significant role in at least the younger children’s ability to plan their movements according to end-state comfort.

## Author Note

We like to thank Alexander Kirmße for his help with conducting this study, all students of the bachelor course “Empirisches Praktikum 2011/2012” for their engagement and their contribution to the implementation of the study design, data collection and coding and Rebecca Schneider also for coding the data. We further thank Roland Pfister and two anonymous reviewers for their constructive and helpful comments on earlier versions of the manuscript.

## Conflict of Interest Statement

The authors declare that the research was conducted in the absence of any commercial or financial relationships that could be construed as a potential conflict of interest.
